# ASO Author Reflections: What Extent of Axillary Surgery Counts? Rethinking Total and Positive Lymph Node Counts After Neoadjuvant Chemotherapy in Young Women with Node-Positive Breast Cancer

**DOI:** 10.1245/s10434-026-20298-9

**Published:** 2026-07-23

**Authors:** Lucy Zhang, Sydney Kinkade, Julie A. Margenthaler

**Affiliations:** Section of Surgical Oncology, Department of Surgery, Washington University School of Medicine in St. Louis, St. Louis, MO USA

## Past

The premise of this study arose from reviewing surgical pathology reports with patients in the office. The number of positive nodes was important, as was the total number of nodes removed, but did that ratio matter? Conversely, for a given ratio, such as 1 of 3 versus 2 of 6, did those counts matter? Thus, the seeds of this study were planted.

Our questions were reshaped and refined over time; ultimately, we drew from several concepts in breast oncology: the aggressive tumor biology of breast cancer in young women, especially human epidermal growth factor receptor 2-positive (HER2+) and triple-negative subtypes susceptible to neoadjuvant chemotherapy; the lymph node ratio (LNR), the proportion of positive lymph nodes to the total number of lymph nodes removed, previously studied across many oncologic contexts; and the ongoing debate surrounding optimal management of the axilla after neoadjuvant chemotherapy in patients with node-positive disease.

This study sought to examine clinical and histopathologic characteristics of a population of young women with node-positive breast cancer who underwent neoadjuvant chemotherapy, and, in particular, the association between recurrence and total lymph node counts across three LNR tiers.

## Present

Overall, as one might expect, those who experienced a recurrence presented with more advanced disease and a greater residual burden of disease after neoadjuvant chemotherapy. Most notably, among patients who had at least one positive lymph node out of at least three nodes excised, there was no significant correlation between the total number of removed nodes and recurrence in any of the three LNR tiers. In other words, we found no difference in recurrence whether there was, say, one positive node of three removed, or 6 of 18.

## Future

Future work should expand on this small, retrospective, single-institution study. Additional subgroup analyses and prospective studies examining larger cohorts over a longer follow-up period are needed. The hope would be to better understand tumor biology and residual tumor burden in the axilla after neoadjuvant chemotherapy; the goal would be to optimize axillary management and guide treatment decisions in both the office and the operating room, especially for young patients with node-positive breast cancer.^1–5^
